# Teaching Competencies in Nursing Professors: Visions of Students and Academics

**DOI:** 10.17533/udea.iee.v41n3e08

**Published:** 2023-10-26

**Authors:** Raúl Quintana-Alonso, Eva García-Redondo, María Miana-Ortega, Elena Chamorro Rebollo, José Antonio Cieza García

**Affiliations:** 1 Registered Nurse, PhD, Associate Dean, Salus Infirmorum Nursing and Physiotherapy Faculty. Pontifical University of Salamanca, Spain. Email: rquintanaal@upsa.es. Corresponding author. Universidad Pontificia de Salamanca Salus Infirmorum Nursing and Physiotherapy Faculty Pontifical University of Salamanca Spain rquintanaal@upsa.es; 2 PhD, Full Professor, Faculty of Education, University of Salamanca, Spain. Email: evagr@usal.es Universidad de Salamanca Faculty of Education University of Salamanca Spain evagr@usal.es; 3 PhD, Full Professor, Salus Infirmorum Nursing and Physiotherapy Faculty. Pontifical University of Salamanca, Spain. Email: mmianaor@upsa.es Universidad Pontificia de Salamanca Salus Infirmorum Nursing and Physiotherapy Faculty Pontifical University of Salamanca Spain mmianaor@upsa.es; 4 Registered Nurse, PhD, Dean, Salus Infirmorum Nursing and Physiotherapy Faculty. Pontifical University of Salamanca, Spain. Email: echamorrore@upsa.es Universidad Pontificia de Salamanca Salus Infirmorum Nursing and Physiotherapy Faculty Pontifical University of Salamanca Spain echamorrore@upsa.es; 5 PhD, Full Professor, Faculty of Education, University of Salamanca, Spain. Email: jacg@usal.es Universidad de Salamanca Faculty of Education University of Salamanca Spain jacg@usal.es

**Keywords:** nursing faculty practice, nursing education research, students, focus groups, qualitative research, práctica del docente de enfermería, investigación en educación de enfermería, estudiantes, grupos focales, investigación cualitativa, prática do docente de enfermagem, pesquisa em educação de enfermagem, estudantes grupos focais, pesquisa qualitativa

## Abstract

**Objective.:**

This work sought to know the view of Nursing professors and students about the competencies the faculty staff must have to deploy their educational function with maximum quality and efficiency.

**Methods.:**

Descriptive qualitative study through focus groups conducted with professors, students and recent Nursing career graduates from universities in Spain.

**Results.:**

The importance of the proposed teaching competencies was delved into, highlighting the importance of professors knowing the context in which they teach, having the ability to self-evaluate their activity, and having adequate interpersonal communication skills, and deploy the teaching-learning process by performing proper planning, using new technologies, and knowing how to engage in teamwork. Moreover, a small discrepancy was detected in relation to disciplinary competence, which students felt was of importance, but which academics indicated is taken for granted in nursing professors; competencies directly related to the act of teaching must be enhanced.

**Conclusion.:**

Practical unanimity was found between academics and students in affirming that the competencies investigated are important for adequate development of the teaching activity in nursing professors. In all cases, the urgent need was highlighted for nursing professors to have adequate teaching training to provide their students with formation of the highest quality.

## Resumen

**Objetivo.:**

Conocer la visión de profesores y alumnos de Enfermería acerca de las competencias que deben presentar los docentes para desplegar su función educativa con la máxima calidad y eficacia.

**Métodos.:**

Estudio descriptivo cualitativo a través de grupos focales realizados con profesores, alumnos y recién egresados del Grado en Enfermería de universidades de España.

**Resultados.:**

Se profundizó en la importancia de las competencias docentes propuestas, destacando la relevancia de que los profesores conozcan el contexto en el que desarrollan la docencia, tengan la habilidad de autoevaluar su actividad, dispongan de unas adecuadas habilidades de comunicación interpersonal, y desplieguen el proceso de enseñanza-aprendizaje realizando una correcta planificación, empleando las nuevas tecnologías y sabiendo trabajar en equipo. Por otro lado, se detectó una pequeña discrepancia con relación a la competencia disciplinar, a la que los estudiantes brindaron una gran importancia, pero que los académicos indicaron que se da por supuesta en los profesores enfermeros, debiendo ser potenciadas las competencias directamente relacionadas con el acto docente.

**Conclusión.:**

Se ha hallado una práctica unanimidad entre académicos y estudiantes en afirmar que las competencias investigadas son importantes para un adecuado desarrollo de la actividad docente en los profesores de Enfermería. En todos los casos, se destacó la imperiosa necesidad de que los profesores de Enfermería dispongan de una adecuada capacitación docente para poder brindar a sus estudiantes una formación de la máxima calidad.

## Resumo

**Objetivo.:**

Conhecer a visão dos docentes e estudantes de Enfermagem sobre as competências que os docentes devem apresentar para desempenhar a sua função educativa com a máxima qualidade e eficácia.

**Métodos.:**

Estudo descritivo qualitativo através de grupos focais realizados com docentes, estudantes e recém-formados da Licenciatura em Enfermagem de universidades espanholas.

**Resultados.:**

Aprofundou-se a importância das competências docentes propostas, destacando a relevância de os professores conhecerem o contexto em que ensinam, terem a capacidade de autoavaliar a sua atividade, terem competências adequadas de comunicação interpessoal e desenvolverem o processo de ensino-aprendizagem através da realização de planejar corretamente, utilizar novas tecnologias e saber trabalhar em equipe. Por outro lado, detectou-se uma pequena discrepância em relação à competência disciplinar, à qual os alunos atribuíram grande importância, mas que os académicos indicaram é um dado adquirido nos professores de enfermagem, devendo ser potenciadas as competências diretamente relacionadas com ato docente.

**Conclusão.:**

Encontrou-se uma unanimidade prática entre acadêmicos e estudantes em afirmar que as competências investigadas são importantes para o adequado desenvolvimento da atividade docente dos professores de Enfermagem. Em todos os casos, foi destacada a necessidade urgente de os professores de Enfermagem terem uma formação docente adequada para poderem proporcionar aos seus alunos uma formação da mais elevada qualidade.

## Introduction

The study of teaching competencies that nursing professors should have to conduct their function effectively and with quality is hardly developed, assuming this fact as an important limitation that not only affects the teacher themselves, who will not have the capacity to adequately transmit to their students the knowledge, skills, and attitudes related to the nursing discipline, but will make it difficult for the students to become competent professionals in the future.^1^^,^^2^ This situation is aggravated because many of the teachers migrate directly from the healthcare field to the academic field without experience or pedagogical training;^3^^,^^4^ in addition, no consensus exists regarding what teaching competencies these professors should develop.^5^^-^^7^ This fact assumes that, often, the training that takes place in Nursing Faculties has a greater relationship with personal motivation or with that teachers saw professors do when they were students, than with a systematized and scientifically endorsed pedagogical process.^8^

Currently, health care is frameworked within a very dynamic setting that must be updated constantly, a fact that causes the disciplinary aspects of nursing to become increasingly complex, requiring from nurses a very high level of theoretical-practical knowledge, as well as the development of skills in communication, empathy, and clinical judgment.^9^^-^^11^ This means that nursing professors must have a high level of training, both disciplinary and pedagogical; such point being one of the most complicated because, today, most professionals who conduct their activity in teaching Nursing have high academic preparation in relation to their clinical area of specialization, but show little formal knowledge in pedagogy and teaching methodology.^12^ Due to all this, it is essential to delve into the study of the teaching competencies professors of the Degree in Nursing must have; the aim of this study was to know the view of Nursing professors and students about the competencies teachers must have to deploy their educational function with maximum quality and effectiveness. 

## Methods

To respond to the objective proposed, a qualitative descriptive study was designed through the development of two focus groups, the first composed by nursing professors and academic authorities and the second by students from third and fourth courses, as well as recently graduated professionals. 

Focus group 1. To develop the first focus group, professors were intentionally selected who represented the reality of teaching in Nursing and who met the following inclusion criteria: (i) Work experience: participants with different years of experience were sought, from 1 year to > 10 years; in cohorts of 1 year, from 2 to 5, between 5 and 10, and > 10; (ii) Gender: a distribution by gender was guaranteed in keeping with the reality of teaching in Nursing; (iii) Specialty: profiles were sought that covered the teaching of the different modules found in Nursing (Basic Sciences, Clinical Sciences, and Psychosocial Sciences); (iv) Responsibility: profiles that held management and/or responsibility positions in the Faculty were included (Deans, Associate Deans, degree coordinator, course coordinators, Department heads, *etc*.); (v) A profile of specialist in pedagogy and/or teaching methodology, outside of Nursing, was selected; and (vi) A profile of person in charge of teacher training programs was sought. The profile of professors and students selected is detailed in Table 1.

**Table 1:** Participants in the teaching focus group

Participant	Gender	Experience	Specialty
P1	Feminine	22 years	Dean
P2	Feminine	1 year	Clinical Sciences
P3	Feminine	15 years	Psychosocial Sciences
P4	Feminine	5 years	Basic Sciences
P5	Masculine	12 years	Education, teacher training manager
P6	Masculine	16 years	Associate Dean

Focus group 2. The second group selected intentionally Nursing students and recently graduated professionals, who met the following inclusion criteria: (i) course: Nursing students with two or more years of university studies (third and fourth courses); (ii) Gender: a distribution by gender was guaranteed that reflected the reality of Nursing students; and, (iii) Graduates in the last promotion at the time of carrying out this phase of the study (2020-2021). The distribution of the participants is shown in Table 2.

**Table 2:** Participants in the focus group of students and graduates

Participant	Gender	Course
A1	Feminine	Fourth
A2	Feminine	Graduate
A3	Feminine	Third
A4	Feminine	Third
A5	Feminine	Fourth
A6	Masculine	Third
A7	Masculine	Third
A8	Feminine	Fourth

In both cases, to guide the development of the sessions, a semi-structured script was drafted. Likewise, the general categories of the study were initially determined, which were designed coinciding with the competencies model proposed, which was formulated after a profound bibliographic analysis about the teaching competencies necessary for professors in the Nursing degree, through which the following competencies were determined:^13^

*1000. Contextual competence:* capacity to incorporate onto the educational practice the essential principles of learning processes that, while justifying the teaching model itself, allow teaching to be placed in the epistemological and sociocultural context of the subject taught.

*2000. Metacognitive competence:* ability to monitor, self-assess, and reflect on one's own pedagogical praxis, determining possible areas of weakness and establishing measures for improvement.

*3000. Planning competence*: capacity to design and develop an academic program, as well as teaching activities and other training resources, adapted to the circumstances of the institution, the profession, and society; selecting for this the most relevant disciplinary content that facilitates the students' learning.

*4000. Methodological competence*: ability to favor and enhance learning and development of personal and professional competencies in students by applying appropriate methodological strategies, according with pedagogical and ethical models adequate to each educational context and situation.

*5000. Evaluation competence:* ability to develop effective strategies to monitor and evaluate acquisition of knowledge and competencies by students, using different instruments in accordance with teaching planning and learning objectives.

*6000. Instrumental communication competence:* ability to develop bidirectional communication processes effectively and correctly, which involves the reception, interpretation, production, and transmission of messages through different channels and media and in a manner contextualized to the teaching-learning situation.

*7000. Interpersonal communication competence:* ability to establish communication processes that promote critical thinking, motivation and confidence in students, recognizing cultural diversity and individual needs, and creating a climate of empathy and ethical commitment with students and colleagues.

*8000. Teamwork competence:* capacity to collaborate and participate as a group member, assuming responsibility and commitment towards tasks and functions assigned to achieve common objectives, following agreed procedures and taking into account available resources.

*9000. Disciplinary competence:* capacity to develop the activities of the Nursing profession with efficacy, quality, and security, demonstrating high level of knowledge and skills related with this discipline.

Development of focus groups of professors and students. Prior to creating the focus groups, participants were contacted directly via telephone to explain the study objectives and the ethical-legal precepts that protected their participation in such, also responding to any questions that might arise about its development. Each participant provided an informed consent, which was signed and returned to the researcher before the start of each session. Given the socio-health situation taking place at the time of conducting the study and with the intention of including participants without geographical distance being a limitation, the focus groups were carried out electronically through the Zoom platform. All sessions were recorded for their later literal transcription. The researcher participated as moderator of the sessions, each lasting approximately 2 h.

Data coding and analysis. In qualitative research, analysis of the data obtained may be considered cyclical and continuous throughout the study;^14^ despite this, the start of this phase can be placed in the literal transcription of the sessions, through initial data interpretation and assignment of meaning to each category. After the literal transcription of the sessions, analysis of the information obtained was structured according to the subprocesses proposed by Miles and Huberman.^15^ Through a data-reduction process, the information was simplified, selecting that which was relevant for the study objectives through an alphanumeric system that allowed coding and organizing the results.

Quality criteria. To ensure the reliability of the results obtained, Lincoln's criteria of credibility, transferability, consistency and confirmability were followed as foundation for the qualitative research.^16^ Moreover, the quality of the analysis of the narratives was verified with some participants selected randomly, ensuring that their thoughts and opinions had been conveyed truthfully.

Ethical considerations. Participation in the study was voluntary and individuals could freely abandon it at any time without reprisal; likewise, no remuneration was foreseen for said participation. Data processing was conducted in keeping with the precepts of Organic Law 3/2018 of December 5, on the Protection of Personal Data and Guarantee of Digital Rights. All recordings were destroyed after their transcription, maintaining the anonymity of the participants during the elaboration of the reports, making sure that no data could be related to a specific individual. The study was approved by the Research Ethics Committee at Universidad Pontificia de Salamanca. 

## Results

As indicated in the previous section, the results will be shown organized in function of the general categories, providing, together with the analysis by the researchers, the discursive fractions that respond most directly to the study’s objective.

### 1000. Contextual competence 3013641498

All the professors manifested the need for teachers to know not only the reality of the discipline in which they teach, but also the structure and operation of the university system, highlighting a frequent situation in nursing professors, who come from the clinical environment to teach at the university, completely ignoring its functioning, an aspect that often causes uncertainty and anxiety: *I believe it is very important to teach professors the context of the university, its bylaws and regulations, its organization. There are many professors who say, I don’t know what I am, if a collaborator, assistant; they do not know what it means, how to achieve it, how their teaching is regulated. It is quite important to teach this context (P5).*

Other professors also exposed the importance of knowing the context of students attending the classrooms, being able to adapt to their reality, which changes with the passage of time: *it is very important to know the context in which we teach and whom we are addressing, for example, currently, with students who are all digital natives, we have to adapt teaching to their reality and to the period we are living (P2).* The students also indicated that it very important for professors to adapt to the context in which they teach, but their perspective differs from that stated by the professors. In this case, there was unanimity among students in stating that professors teaching in the Nursing career and who are not nurses should know the context of the discipline in which they teach to, thus, adapt the teaching load of their subjects to what is really necessary for professionals of this nature: *I believe there are professors who, by not being in nursing, take their subject as if we were in that career and we have to study as they studied, we have to know as they know and sometimes you have to say, let’s see, I am in nursing, I don't have to downplay the importance of my subject, but they study to be nurses (A3).*

### 2000. Metacognitive competence

For the participants in both focus groups, metacognitive competence had much importance, focusing as a priority on the self-evaluation teachers should perform of their teaching activity as a learning element and improvement for them: *independent of external evaluation processes, which I agree are necessary, professors must continuously ask: “am I doing it well? How can I improve? (P4).* In this sense, the idea also emerged that many teachers are reluctant to assess their performance, either because of difficulty in assuming that they may have room for improvement or because they do not consider that they should change anything: *I feel that perhaps professors do not assign much importance to the evaluation of their teaching because they do not like to be told what they do wrong; that is often difficult to accept (P3); I believe there is a part of teachers themselves that has to assume that perhaps they lack some competence or skills. (…)many professors find it hard to accept that they can be mistaken in that sense (A2)*.

Finally, In the group of professors, the importance of participating in external evaluation processes such as Docentia was highlighted, manifesting the need for professors to know their functioning and participate in such: *I do believe it is important to know about and participate in these evaluation processes, for example Docentia. It helps you stop and reflect on what you have been doing for the last four years, how you have been teaching classes, what training you have undergone and that helps you to detect areas of improvement and be able to remedy them (P1).*

### 3000. Planning competence

Professors and students stated that teaching planning is one of the fundamental elements for success in achieving the teaching-learning objectives: *the planning competence is very important, nothing can be taken for granted and,* f*or this reason, class dynamics must be organized and designed to know where we are starting from and where we want to go (P2).* In addition, both groups highlighted that one of the fundamental elements to plan teaching adequately is that of determining the level of students’ prior knowledge, being the element upon which to base subsequent teaching development: *I think that in certain assignments, for example, in Clinical Nursing, which deals with aspects of physiology, anatomy, and pathology, it is super important to know the students’ level of knowledge because they might not remember anything from physiology and we must start again from there, if not, they won’t learn anything (P1); furthermore, that gives us much more security (…) It has been seen, for example, in other assignments, for example, in English no prior evaluation was made and we saw what happened, the whole class failed, then the methodology changed and it went well (A3).*

### 4000. Methodological competence

Regarding methodological competence, it was exposed that, although it is true that traditional methodologies, like master classes, continue being highly important in the teaching-learning process, it is necessary to include teacher innovation tools that adhere to trends in technological progress characteristic of today's society: *I consider that the master class continues being a valid methodology that works, but it is true that we also have to do innovative things, use new methodologies that make students participate more (P1).* For the students, the use of practical, visual, and digital methods assumes their increased attention and motivation in the classes, thereby, they advocate for professors increase said use in teaching, especially in disciplines, like Nursing with high practical activity: *teaching should be more practical. If a procedure is going to be explained, it makes no sense to do it with slides. Give me the information, but let me see how it is done and practice doing it because then we study the procedure and go to the hospital and see that it really has nothing to do with what we were explained (A6); visual methods contribute much and favor much when learning and make the class less boring and more enjoyable (A8).*

### 5000. Evaluation competence

One of the most important functions for teachers is that of knowing how to organize evaluation tools that objectively permit determining if students have acquired the knowledge and competencies established for each assignment, thus, this competence is considered quite important by teachers and students participating in the focus groups. From the students’ point of view, it is important for teachers to plan adequately evaluations, highlighting two aspects: that they are previously informed of the evaluation methodology and that such represents an intellectual challenge for them: *a bad way to evaluate is to provoke studying by rote memorization and vomiting notes (A3); if professors want to request certain things from you to evaluate you, they need to make it clear to you, that is the important thing, rather than then giving you X things on the exam so that you can demonstrate what you know. If a person makes it clear what is asked of you, you should have no problems getting to the exam and complying with that evaluation. (A7).*

In this sense, professors stated that the evaluation is one of the most difficult functions for any teacher, being it important to design strategies that not only permit assessing knowledge, but the students’ acquisition of competencies; an element in which more information would be necessary. Additionally, they considered it very relevant to have knowledge to be able to assess their evaluation: *regarding the evaluation by competencies, it is one of the issues feared most by professors because, for example, how do you evaluate biochemistry through competencies? It is what agencies ask us to do and we have to do it, but I think we don't know and it's a point in which they have to teach us (P6).*

### 6000. Instrumental communication

It is interesting that professors and students indicated that instrumental communication is not as relevant for teachers, despite being the one that enables them to express themselves adequately via scientific-technical language and to use verbal and non-verbal resources for proper class development, placing the focus of communication on the interpersonal setting: *Adequate technical level in the language of teachers is assumed, which is why the focus should be on their acquiring more interpersonal skills than instrumental ones in their communication (P3); we prefer a professor who treats us well in class, who is concerned about us, than someone who goes through everything but speaks very well and uses a lot of technical language (A4).*

### 7000. Interpersonal communication

Both in the focus group with students as with professors, the idea emerged with intensity that interpersonal communication is one of the most important competencies that must be present in a teacher, especially in careers, like Nursing, where treatment with others, generally in situations of sickness and vulnerability, is one of the characteristic aspects of their daily exercise, which should be taught to students, not only from theory, but with their professors’ examples: *It seems to me that interpersonal communication is very important because teaching is not only giving a topic, but also knowing the students, knowing that what you are delivering reaches them and that you are motivating them (A8); I feel it is a fundamental competence to be a good teacher because students come with their particular circumstances and these are not always favorable for learning, then, giving them the confidence to open up at some point, to share their situations; that part of relationship with students I believe is very important (P4).*

### 8000. Teamwork competence

With regards to teamwork competence, all the participants stated that professors must have this competence, especially when teaching subjects together with other teachers, given that lack of coordination between them directly impacts on the quality of the classes and the students' satisfaction with them: *I consider it fundamental to know how to work as a team, to know how to coordinate faculty professors, but most important in Nursing it is to know how to include expert clinical nurses who provide their hospital vision. Either the teaching staff knows how to work together or students notice it quickly and trust and credibility are lost (P3); that is noticed, when professors from a given assignment talk and communicate with each other for students to receive the best. I know people from other universities who have 10 professors in an assignment, this one has taught me this, this one repeated it again, this other one did not know what the others had taught… and in the end that is bad for students; that is why, organization among professors is fundamental (A3).*

### 9000. Disciplinary competence

At this point, a slight discrepancy was observed between the professors and the students. The first stated that disciplinary competence is not as important, given that nurse professors must have prior knowledge related with their discipline, therefore, the focus must be on the competencies necessary to transmit said knowledge appropriately to their students: *if we continue thinking that disciplinary competence is most important, we leave planning, methodological, etc., competencies relegated. That is, We join teaching because we are experts in the clinical area, but then the planning and methodological part, which for a teacher are more important, we leave aside (P2).*

However, the students did consider this competence more important, claiming to have found substantial differences in the classes taught by nurse professors with greater experience: *even professors must have noticed differences between one another (A3); experience also allows you to teach it to another person. For you, experience has been able to facilitate that problem that you had in your day and now it helps you teach it to other people. (A4).*

## Discussion

University professors must be qualified professionals for theoretical, profound, and critical analysis of educational phenomena related with their discipline. This fact will allow them to design the context, policies and teaching processes, creating optimal conditions not only for the academic development of their students, but also for their moral and personal growth.^17^ To carry out their work, it is necessary to develop adequate teaching competencies, especially when university professors - particularly in in health sciences - usually enter teaching without prior pedagogical or didactic training, migrating from other professional spaces, mainly healthcare activity, an aspect that must undoubtedly limit the educational activity carried out.^18^

Within the context of Nursing sciences, few studies have addressed the competencies their professors must have,^2^^,^^19^ being models focused mainly on pedagogical and disciplinary skills, but which have not contemplated other fundamental elements, like the metacognition of the teaching function and knowledge of the context in which it is carried out, a fact that causes the competencies analyzed in this study to represent the most complete model published for nursing professors.

Regarding these two competencies mentioned, their inclusion in the model responds to their importance for the quality of teaching. In this respect, evidence consulted affirms that reflexive teaching practice, included in metacognitive competence, is one of the most important processes in teacher training, given that it stimulates teachers to develop different skills, such as introspection, decision making, and logical thought.^20^ This reflection means that teachers maintain reflexive thinking that encourages them to review their experience in the classroom systematically and cyclically, using their perceptions and experiences, as well as the evaluations of their students, to assess the quality of their performance, taking actions to improve their teaching and their students’ learning standards.^21^ In turn, in relation to contextual competence, other publications indicate that educational centers represent a complex multidimensional environment, where political, social, organizational, personal, and academic aspects are interrelated, which impact directly on the way professors conduct their activity, as well as their students’ learning, with this situation being expressed by many novel professors as a “reality shock”, manifesting that often they have not received training about the context in which they have to carry out their teaching, representing an important barrier during the start of their teaching career, which impacts upon the quality of the education they provide to their students.^22^

Regarding methodological competence, it is imperatively necessary for teachers to not only have adequate training, but also a methodological culture, which allows them to deploy the teaching-learning process with the highest quality, thus, providing their students with the knowledge and competencies necessary to acquire their maximum potential in the professional and personal levels.^23^ Moreover, communication by a professor in the classroom is a key aspect, with authors stating that the teaching-learning process can be reduced to a communicative process whose purpose is to promote acquisition of knowledge, skills, and attitudes by students. In this sense, the fact that interpersonal communication was considered more important than instrumental communication can be related to what was stated by other studies, which indicate that when students feel accepted by professors these have greater motivation, confidence, and predisposition in classes, besides a better mood during such, even improving their academic performance.^24^

With respect to teamwork, the importance assigned to this competence is in line with information published in other studies, which claim that settings in which teachers have this competence improve effectiveness when conducting their tasks, established objectives are achieved to a greater extent and there is a better predisposition to solve problems.^25^

Finally, about disciplinary competence, it is highlighted that despite the views of professors in relation with its relative importance, an adequate level in this competence must be ensured, given that it refers to knowledge about the nursing discipline that must be transmitted to students, being, therefore, included in all the models of teaching competencies for nursing professors found in the scientific literature.^6^^,^^7^^,^^19^

Practical unanimity has been found between academics and students in affirming that the competencies researched for adequate development of the teaching activity in nursing professors. Further, discrepancy was detected in relation to disciplinary competence, to which students gave great importance, but which academics indicated that this is taken for granted in nurse educators, with competencies related directly with the teaching act having to be enhanced. In all cases, the urgent need for nursing professors to have adequate teaching training to provide their students with training of the highest quality was highlighted.
